# Assessment of a novel Ehlers-Danlos syndromes disability index

**DOI:** 10.3389/fresc.2024.1280582

**Published:** 2024-04-19

**Authors:** Stephen Chai, Patricia Roney, John Fagan, Emily Rose Rosario

**Affiliations:** Research Institute, Casa Colina Hospital and Centers for Healthcare, Pomona, CA, United States

**Keywords:** Ehlers-Danlos syndrome, EDS, patient reported outcomes, hypermobility, pain management, disability index

## Abstract

**Background:**

The Ehlers-Danlos syndromes (EDS) are a group of inherited connective tissue disorders characterized by disruptions in collagen synthesis and processing. These disorders lead to various symptoms, including hypermobility, musculoskeletal conditions, and chronic pain that can significantly limit patients' daily living. In the absence of a curative treatment, an EDS specific disability index that tracks changes in patient-reported outcomes can facilitate the investigation of new treatment options and enhance the quality of life for EDS patients.

**Methods:**

An EDS-specific disability index was created using survey data and input from clinicians. A total of 222 EDS patients in a multidisciplinary clinical program completed the index during their initial visit. Exploratory and confirmatory factor analyses were conducted to determine the index's factor solution and assess its goodness-of-fit. Paired *t*-tests were performed with follow-up visit data collected over the course of one year.

**Results:**

The exploratory and confirmatory factor analyses indicated a two-factor solution, accounting for 42.40% of the variance. The index demonstrated adequate fit to the data, supported by Tucker and Lewis's index (0.85) and root mean square error of approximation (0.1). Data from follow-up visits showed significant improvement in three symptom related variables and one function related variable in addition to the total score and the symptom subscale score when compared to the initial visit.

**Conclusion:**

The development of an EDS-specific disability index is a crucial step in creating a clinical tool that enables healthcare professionals to gain a deeper understanding of the impact EDS has on patients’ lives and potentially identify new therapeutic interventions.

## 1 Introduction

Chronic musculoskeletal disorders negatively affect health-related quality of life and have a significant impact on both direct healthcare utilization and indirect productivity costs (1, 2). Among these disorders, Ehlers-Danlos Syndromes (EDS) deserve greater attention from healthcare professionals and systems. EDS are a diverse set of heritable disorders of connective tissue that commonly present as joint hypermobility, skin hyperextensibility, and tissue fragility (3). Patients with EDS often experience a range of symptoms as these disorders involve multiple systems, from joint and skin to functional gastrointestinal disorders, fibromyalgia, fatigue, cognitive issues, sleep disturbances, allergies, and migraine headaches (4–6). The clinical presentation of EDS is complex, influenced by genetic variations and inconsistent timing of symptoms. Consequently, patients with the same disease can display diverse symptoms, presenting challenges in both identification and treatment. Recent studies have highlighted this difficulty, reporting on the marginalization of EDS patients who commonly face delayed diagnosis, misdiagnosis, and inappropriate treatment and report feelings of distrust and insecurity (7, 8). The most common type of EDS is hypermobile EDS (hEDS), similar to hypermobility spectrum disorder (HSD) though with different diagnostic criteria. The diagnosis often involves meeting specific criteria set forth by medical guidelines, such as the 2017 International Classification of the Ehlers-Danlos Syndromes. This includes features such as joint hypermobility, skin hyperextensibility, and a family history of similar symptoms (3).

Therapeutic measures to treat EDS are often multidisciplinary, depending on a combination of several different treatments for optimal symptom control (9). Specifically, physiotherapy has been consistently reported to have a positive impact on EDS patients' quality of life. However, the absence of uniform outcome measures, particularly in assessing functional capacity has hindered cross-study comparison (10). Patient-reported outcome measures (PROMs) are a valuable tool for monitoring the progression of symptoms and evaluating the effectiveness of treatment options (11, 12). By collecting information directly from patients about their health status or treatment, PROMs provide a unique perspective that is not influenced by clinician interpretation (13, 14). Thus, a disease specific PROM that can identify interventions positively affecting the various dimensions of disability that EDS patients face is crucial given the absence of a curative treatment. Currently, the Bristol Impact of Hypermobility (BIoH) stands out as the most extensively validated PROM for hypermobile EDS populations. With its maximum score of 360, the BIoH encompasses domains such as pain, fatigue, physical function, anxiety, planning and management, as well as strength and weakness. While the BIoH has demonstrated strong construct validity and test-retest reliability, it is important to acknowledge a few limitations in its methods and applicability (15, 16). In its initial validation, the diagnosis of joint hypermobility syndrome in participants was self-declared and varying item maximum scores hindered factor analysis. Therefore, in this study we created an EDS Disability Index that could be validated through exploratory and confirmatory factor analyses to identify underlying disability dimensions, enabling targeted treatment in the future. We report initial findings in assessing the validity and sensitivity of our EDS Disability index towards augmenting our overall understanding of EDS and becoming a clinical decision-making tool for evaluating the efficacy of therapeutic interventions.

## 2 Method

### 2.1 Design

This study was approved by the Research and Human Subjects Review Committee at Casa Colina Hospital and Centers for Healthcare (CCH). Participants included individuals in a multidisciplinary clinical program for EDS at CCH that provides a care continuum extending from medical-surgical care to acute rehabilitation and outpatient therapies. The diagnostic process is based on the 2017 International Classification of the Ehlers-Danlos Syndromes (3) and involves a thorough evaluation of medical history, physical examination, and genetic testing to identify less common types of EDS. The majority of participants have a diagnosis of hEDS. Once a diagnosis is confirmed, the physician specialist collaborates with patients' primary care doctors to create a personalized treatment plan. The EDS therapy team tailors the treatment plan to patients' specific needs, utilizing safe and effective exercises such as light weight training, isometric holds, and the option for aquatic therapy. The focus is on building strength, increasing endurance, protecting joints, and improving stability, all aimed at enabling patients to resume activities they enjoy. CCH staff organize regular follow-ups to ensure ongoing effectiveness of the treatment plan, often involving physical and occupational therapy. Additionally, a monthly EDS support group provides emotional support, community, and valuable educational information.

### 2.2 Index structure

**Figure 1:**
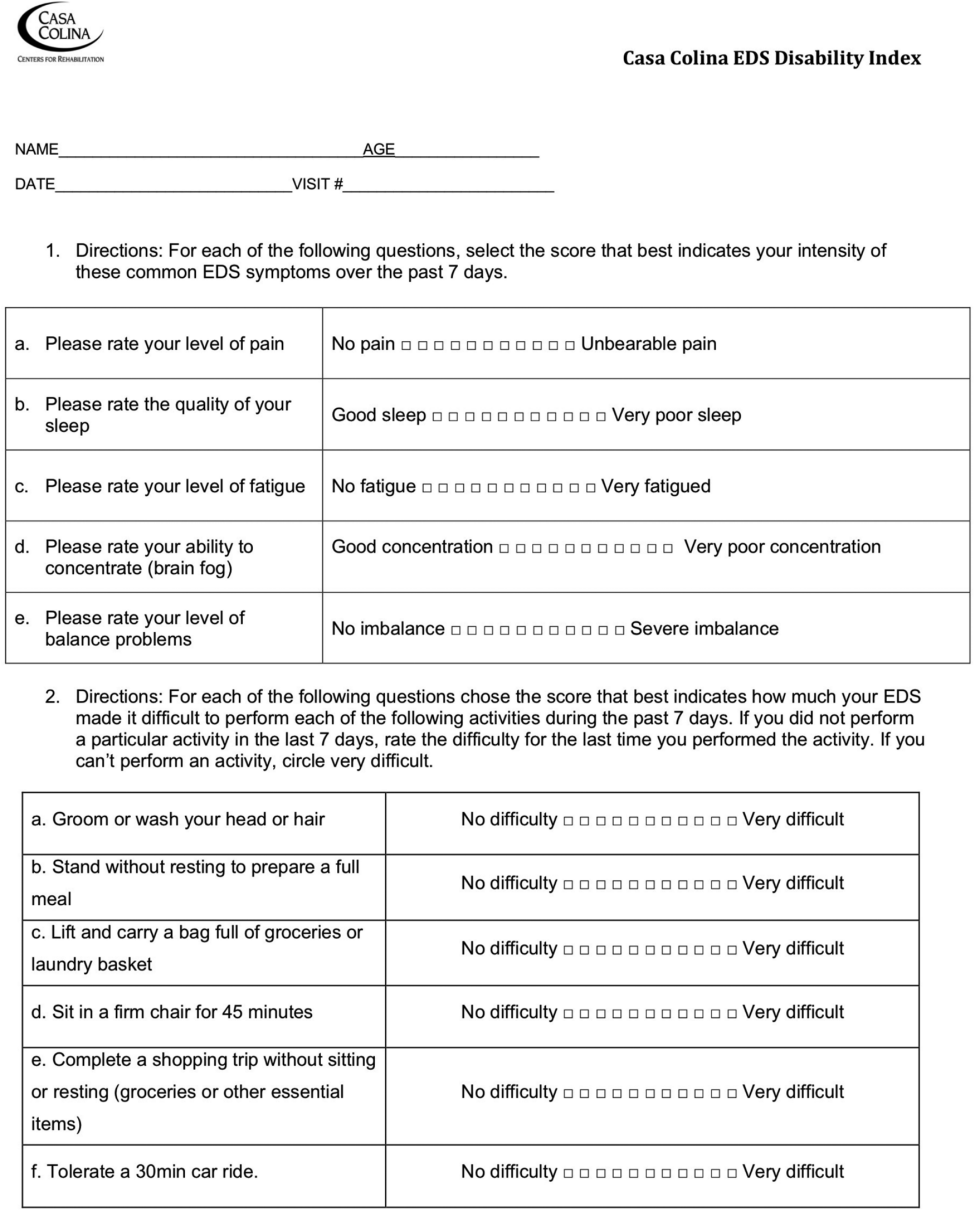
Casa Colina EDS Disability Index.

The disability index was created with survey data that had been collected over one year. Therapists with experience working with EDS patients were consulted to review the data and identify impactful questions for the study. Clinicians additionally provided input to further ensure the selected questions were pertinent for the patient population based on their experience. Initially, the index consisted of 16 questions that required participants to rate their EDS symptoms and functional abilities on a scale of 1–10. Following exploratory factor analysis, the index was refined to 11 questions (Figure 1), considering factor loadings and goodness of fit analyses. This structure facilitates an understanding of both the effectiveness of a patient's treatments and the impact of EDS symptoms on their quality of life.

### 2.3 Analysis

A total of 222 EDS patients completed the index, and the date of completion was marked as their initial visit. Our participant group comprised 90% females and 10% males, with an average age of 32.4 years (±15.2) and an average number of visits of 9.6 (±10.8). To assess the validity of our newly created index, the collected data was subjected to exploratory and confirmatory factor analyses, as well as goodness of fit analysis. An exploratory factor analysis was performed to determine the potential number of factors underlying the index. This involved retrieving eigenvalues, which were plotted on a Scree plot for visualization. A confirmatory factor analysis was then performed to confirm the factor solution with a likelihood ratio test, the total variance accounted for by structure, and the goodness of fit indices. Both the Tucker and Lewis's Index (TLI) and Root Mean Square Error of Approximation (RMSEA) were used to evaluate the model's goodness of fit. The RMSEA ranges from 0–1, with lower values indicating better model fit. An RMSEA value of.06 or less is indicative of acceptable model fit, while for TLI, values closer to 1 indicate better fit, with values above. 95 indicating good fit (17). To facilitate model comparison the Akaike (AIC) and Bayesian Information Criterion (BIC) were also presented, where smaller values indicate better fit. To refine the initial index, items were removed if their factor loading was less than 0.3 (18). Subsequently, paired *t*-tests were conducted on return visits for each reported outcome using the refined index and factor summations to assess its reliability over time.

## 3 Results

### 3.1 Exploratory factor analysis

**Figure 2:**
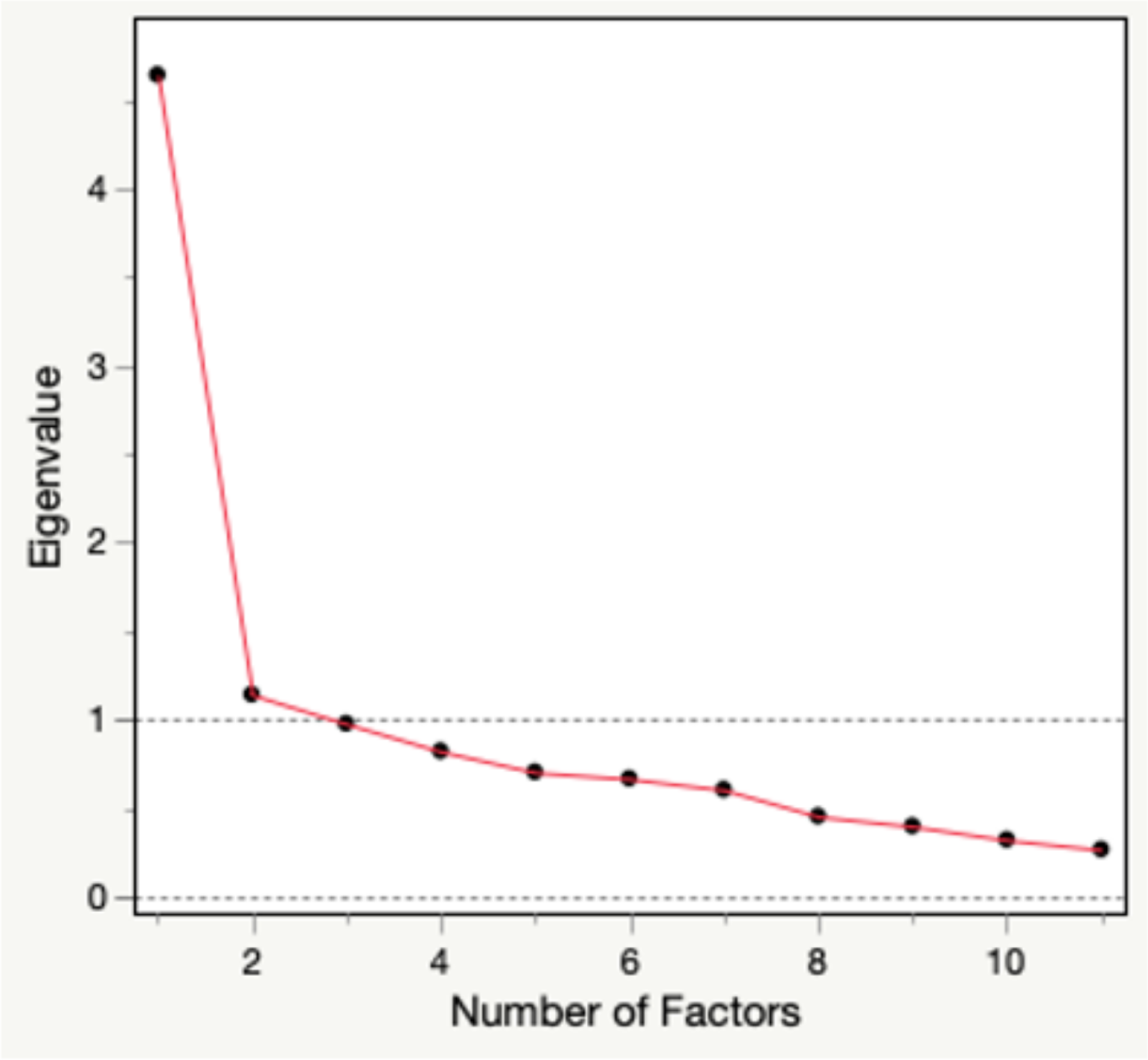
Scree plot.

The exploratory factor analysis yielded a two-factor solution. The first two eigenvalues were 4.64 and 1.14 while the subsequent eigenvalues were less than 1.0 (Figure 2). The factor loadings ranged from 0.30–0.81. All variables loaded onto the two factors.

### 3.2 Confirmatory factor analysis

The likelihood ratio test (Prob > chisq 0.0001, DF = 55, ChiSquare = 852.235, Table 1) indicated that a two-factor structure sufficiently explained the data variance and accounted for 42.40% after rotation. Table 1 additionally displays the goodness of fit indices indicating an adequate model fit: AIC (41.745), BIC (−73.638), TLI (0.852) and RMSEA (0.101). Based on the rotated factor loadings, the two latent factors were identified as “clinical symptoms” and “functional activity” and demonstrated an average variance of 0.48 and 0.55 respectively.

**Table 1:** Significance test and measures of fit.

Test	DF	Criterion	ChiSq	Prob > ChiSq	Measures of fit			
					AIC	BIC	TLI	RMSEA
H0: 2 factors are sufficient	34	0.501	106.822	0.0001*	41.75	−73.64	0.85	0.10

DF, degrees of freedom; ChiSq, chi-square; AIC, akaike information criterion; BIC, bayesian information criterion; TLI, tucker and Lewis's index; RMSEA, root mean square error of approximation.

* *p* < 0.05.

The paired *t*-test analysis revealed a significant change in the total score as well as improvements across various PROMs between the initial and follow-up visit (Table 2) (*p* < 0.05), including symptom related variables such as sleep, fatigue, and concentration and the function related variable of sitting in a firm chair for 45 min. Additionally, when examining changes in subscale scores for symptoms and functional activities, the paired *t*-test for the symptom subscale score demonstrated a notable improvement (*t* = 3.2, DF = 106, *p* = 0.002).

**Table 2:** Paired *t*-test with revised EDS index.

	Initial visit	Follow up visit	Paired *t* test		
	(Mean ± SD)	(Mean ± SD)	*T* value	DF	*P*=
Pain	5.0 ± 2.0	4.7 ± 2.1	1.3	106	0.21
Sleep	5.8 ± 2.4	5.1 ± 2.6	2.6	106	0.01*
Fatigue	6.6 ± 2.0	6.0 ± 2.3	2.8	106	0.006*
Concentration	5.8 ± 2.4	5.1 ± 2.2	3.1	106	0.002*
Balance	4.1 ± 2.3	4.0 ± 2.1	0.6	106	0.53
Wash head/hair	3.4 ± 3.0	3.3 ± 2.9	0.5	106	0.59
Stand to prepare meal	5.8 ± 4.7	5.7 ± 2.7	0.2	106	0.87
Carry bag of groceries	5.3 ± 3.0	5.1 ± 2.6	1.0	106	0.31
Sit in firm chair for 45 min	6.1 ± 3.0	5.6 ± 2.7	2.4	106	0.02*
Shopping trip without sitting	5.2 ± 3.1	4.9 ± 3.1	1.2	106	0.24
Car Ride for 30 min	3.5 ± 3.0	3.4 ± 2.7	0.4	106	0.7
Total Score	55.9 ± 20.6	52.8 ± 18.5	2.0	106	0.04*
Symptom subscale	27.1 ± 7.8	24.9 ± 8.2	3.2	106	0.002*
Functional subscale	28.7 ± 15.1	27.9 ± 12.3	0.7	106	0.47

SD, standard deviation; DF, degrees of freedom.

* *p* < 0.05.

## 4 Discussion

Our study marks the first step in the development of a novel, disease-specific disability index for EDS patients, addressing a crucial gap in the field. Our initial analysis of the index supports a two-factor solution that demonstrates an adequate model fit, as evidenced by various indices. The index demonstrated that it was responsive to change over time through a significant total score change as well as potential sensitivity to treatments that affect certain subdomains of health. Assessing changes in different subscale scores allows for a more in-depth evaluation of the overall impact of a particular treatment or intervention. This can help clinicians and researchers to better understand the needs of patients with disabilities and to develop more effective interventions that address a range of different challenges.

Future directions should involve measures that further refine the index. Currently, the EDS index lacks measurement of the social domain of health. Recent studies have shown that components of emotional health are often failed to be addressed in EDS patient's management (19). To strengthen the index's comprehensiveness, adding questions that target commonly reported social domains of health like anxiety and depression could be beneficial. To identify appropriate PROMs for inclusion, comparisons can be made between the RMSEA, TLI, AIC, and BIC values of updated indexes and those presented in this study. Furthermore, variables showing consistent unresponsiveness to treatments require careful evaluation. A few variables in this study did not reveal significant pre-post changes. Factors such as timing of assessment post-treatment and variations in patient populations should be considered as they can impact responsiveness. Therefore, longitudinal studies and testing the index on diverse patient cohorts are necessary before labeling a measure as insensitive. However, if certain variables consistently prove insensitive, discussions should shift towards their potential removal.

### 4.1 Limitations

Our study is not without limitations. First, the study sample had a higher proportion of a of hEDS. Although the classic and hypermobile subtypes are the most common subtypes of EDS out of the 13 recognized by the International EDS Consortium, the absence of a distribution of EDS types in our patient cohort limits the generalizability of our findings to other populations. Numerous patients were referred to the program solely based on hypermobility, a broadly shared trait among several of the previously mentioned EDS subtypes. Specifying the exact subtypes that EDS patients present with could enhance index specificity. Additionally, we did not report on the presence of comorbidities in our population. It is not uncommon for EDS patients to present with gastrointestinal functional disorders, asthma, mast cell activation syndrome, and postural orthostatic tachycardia syndrome (20, 21). The presence of these comorbidities might have led to patients undergoing various treatments and surgical interventions, potentially affecting their response to physical therapy, and introducing confounding variables. Identifying the presence of these comorbidities in EDS patient populations should be considered in the future, as their underlying mechanisms have received increasing attention in the last decade. Our study also did not account for variations in treatment types and intensities among participants during follow-up. Tailored treatments could influence patient outcomes differently, potentially resulting in varied improvements. It is important to consider this limitation when reviewing the visit numbers as well, as individuals new to the clinic may experience greater changes compared to those who have already made baseline improvements. Another limitation of our study is the middle age range reported for the participants. PROMs for children with EDS may differ from those of older patients, as their management and perception of pain can differ (22). Recent studies on the use of various functional outcome measures for adolescents with hypermobility spectrum disorder lack an EDS-specific evaluation tool (23, 24). Furthermore, initial assessment of the index measures did not include evaluating reliability and stability. Assessing test-retest reliability in outcome measures is crucial to ensure consistent scores over time, particularly in patients reporting stable conditions (25). The decision to prioritize treatment assessment and factor analysis was based on the recognition that the EDS patients were at different treatment stages. Improvement in scores over a two-week period, the timeframe used in the BIoH questionnaire for assessing test-retest reliability, was anticipated. Future efforts should incorporate test-retest reliability assessment, particularly in patients presenting to the clinic for the first time, to demonstrate reliability and stability. Finally, the items in this scale do not include mental health variables. We fully agree that the mental health aspect of EDS, such as anxiety and depression, plays a significant role in patients’ overall well-being and disability. While our initial index may have overlooked these critical aspects, we acknowledge the need to incorporate measures to assess mental health outcomes comprehensively. Future iterations of the disability index will look to include these items.

## 5 Conclusion

Our preliminary findings further the development of a patient-reported disability index for EDS patients that comprehensively encompasses the various dimensions of disability they encounter. This index has great potential to improve our understanding of the disease and guide the creation of novel therapeutic interventions. However, it requires further development and testing before widespread clinical use by healthcare systems and professionals.

## Data Availability

The original contributions presented in the study are included in the article/supplementary materials, further inquiries can be directed to the corresponding author.
