# Corrigendum: Assessment of a novel Ehlers-Danlos syndromes disability index

**DOI:** 10.3389/fresc.2024.1430453

**Published:** 2024-05-17

**Authors:** Stephen Chai, Patricia Roney, John Fagan, Emily Rose Rosario

**Affiliations:** Research Institute, Casa Colina Hospital and Centers for Healthcare, Pomona, CA, United States

**Keywords:** Ehlers-Danlos syndrome, EDS, patient reported outcomes, hypermobility, pain management, disability index

A Corrigendum on Assessment of a novel Ehlers-Danlos syndromes disability index By Chai S, Roney P, Fagan J, Rosario E (2024). Front. Rehabil. Sci. 5:1280582. doi: 10.3389/fresc.2024.1280582

**Text Correction**

In the published article, there was an error. Some contributions were missing from the Acknowledgments statement.

A correction has been made to **Acknowledgments**. This statement previously stated:

We would like to express our thanks to the Casa Colina Board of Directors and CEO Kelly Linden for their support of the Casa Colina Research Institute. We also extend our thanks to the Casa Colina Foundation for their generous funding, which has enabled us to make significant strides in this field. Lastly, we thank all the participants of this study for their invaluable contributions, without which this research would not have been possible.

The corrected Acknowledgments statement appears below:

We would like to extend our gratitude to the Casa Colina Board of Directors and CEO Kelly Linden for their unwavering support of the Casa Colina Research Institute. The generous funding provided by the Casa Colina Foundation has played a vital role in advancing our research efforts. We are also deeply thankful to all the participants of this study for their invaluable contributions.

Furthermore, we wish to acknowledge the substantial contributions of Rachel Yaghoubian and Sarah Barnes to the manuscript. The inception of the EDS Disability Index stemmed from their discussions regarding the lack of appropriate outcome measures for individuals with EDS. Through collaboration with the EDS Program team, they recognized the necessity for a more streamlined patient-reported outcome measure (PROM). Their collaborative endeavors have been instrumental in the development of the EDS Disability Index.

The authors apologize for this error and state that this does not change the scientific conclusions of the article in any way. The original article has been updated.

